# Stump appendicitis, a case report and a review of the literature. Is it as uncommon as it is thought?

**DOI:** 10.1016/j.ijscr.2020.02.016

**Published:** 2020-02-11

**Authors:** Daniela Burbano, Alberto Federico García, Julián Chica Yantén, Camilo Salazar, Juan Sebastian Toro, Juan Carlos Bravo

**Affiliations:** a School of Medicine, Universidad ICESI, Calle 18 No. 122-135, Carrera 98 No. 18-49, Cali, Colombia; b Center for Clinical Research (CIC), Fundación Valle del Lili, Carrera 98 No.18-49, Torre 7, Piso 3 (CIC), Cali, Colombia; c Department of Surgery, Fundación Valle del Lili, Carrera 98 No. 18-49, Cali, Colombia; d Department of Radiology, Fundación Valle del Lili, Carrera 98 No. 18-49, Cali, Colombia; e Department of Pathology, Fundación Valle del Lili, Carrera 98 No. 18-49, Cali, Colombia

**Keywords:** Stump appendicitis, Abdominal pain, Recurrent appendicitis, Appendiceal pathology

## Abstract

**Introduction:**

Acute appendicitis (AA) is the most common cause of acute surgical abdomen. Complications from surgical appendectomy include intraabdominal abscess, bleeding, surgical site infections, ileus, and stump appendicitis (SA). This last one is one of the least common ones with a reported incidence of 1:50.000.

**Methods:**

We present a case and review 132 cases of SA reported in the literature. Demographic and clinical characteristics were evaluated, as well as details of the surgical treatment. Categorical variables are presented as quantities and proportions, and continuous variables with median and interquartile range. Additionally, we calculate an incidence from 3 papers reported in the literature and our own.

**Results:**

We analyzed 132 cases, 60.3 % were male with a median age at SA of 33 years. There was a wide range time interval between the episode of AA and SA from 1 day to 60 years. Initial open appendectomy was reported in 62 cases. From all the patients with SA 51 % reported complications. The median length of the appendiceal stump was 3 cm. The incidence of SA fluctuated between 0.22–1.37 in 1.000 cases of appendectomies.

**Discussion:**

SA is usually underrated, and which are the risk factors for this condition are not clear. The data available suggest that a primary laparoscopic appendectomy is not related to SA, but the length of the stump left in the first surgery might be associated. The incidence of SA seems to be higher than the one reported of 1 in 50.000.

## Highlights

• The clinical presentation of stump appendicitis is similar as a first episode of appendicitis.
• The risk factors for SA are not clear, but the data available suggest that a primary laparoscopic appendectomy is not related.
• The length of the stump left in the first surgery might be associated with stump appendicitis.
• Incidence of stump appendicitis seems to be higher than previously reported.

## 1 Introduction

Acute appendicitis (AA) is the most common cause of acute surgical abdomen (38.9 %) [1,2]. Complications are more common when AA is associated with perforation [3,4], and they include intraabdominal abscess formation, bleeding, surgical site infections, ileus, fistulas, and stump appendicitis (SA) [[5], [6], [7]]. SA was first described in 1945 [7] and is defined as the inflammation of the appendiceal remnant after a surgical appendectomy [8]. Its reported incidence is of 1:50.000 even though this number does not have support.

## 2 Methods

We present a case report of SA. Additionally, we performed an electronic search for case reports using the terms “stump appendicitis,” “residual appendicitis,” or “retained appendix” in PubMed, LILACS, Scopus, Embase, and ScienceDirect. The search was limited to articles published in English, Spanish, and German. We identified 97 articles that provided 131 cases. We added our case for a total of 132 cases. The data was analyzed with Stata 15.1®, (College Station, TX). Demographic characteristics were evaluated, categorical variables are presented as quantities and proportions, and the continuous variables with mean and standard deviation (SD) or median and interquartile range (IQR). Furthermore, we calculated the incidence of SA of three articles plus, our own.

The work has been reported in line with the SCARE criteria [9].

## 3 Case report

**Fig. 1:**
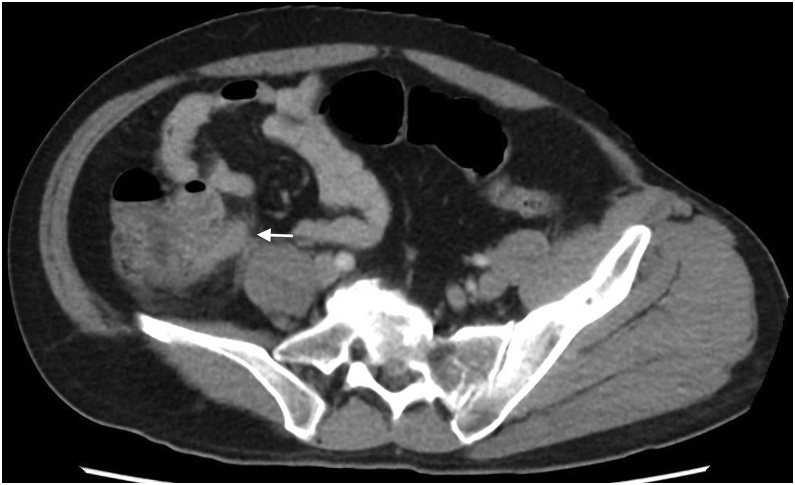
Tubular image suggestive of appendicular stump with edema.

A 49-year-old male patient presented to the emergency room with colicky abdominal pain over the last ten days localized in the right lower quadrant (RLQ), associated with abdominal distension, vomiting, and constipation. As a past medical history, he had an open appendectomy at childhood and had suffered RLQ pain in the last couple of years. At admission, he was afebrile, with normal vital signs, abdominal tenderness, and doubtful Blumberg sign. Laboratory exams showed leukocytosis, neutrophilia, and elevated C reactive protein. Contrast computed tomography (CT) of the abdomen and pelvis showed an appendiceal stump of 1.3 cm long with inflammatory signs (Fig. 1). With this data, an exploratory laparoscopy was indicated. During this procedure, dense adhesions in the ileum and cecum and a lump in the cecum hindered the surgical procedure. It was converted to an open procedure. A segmentary ascending colectomy with ileocolic anastomosis was performed.

**Fig. 2:**
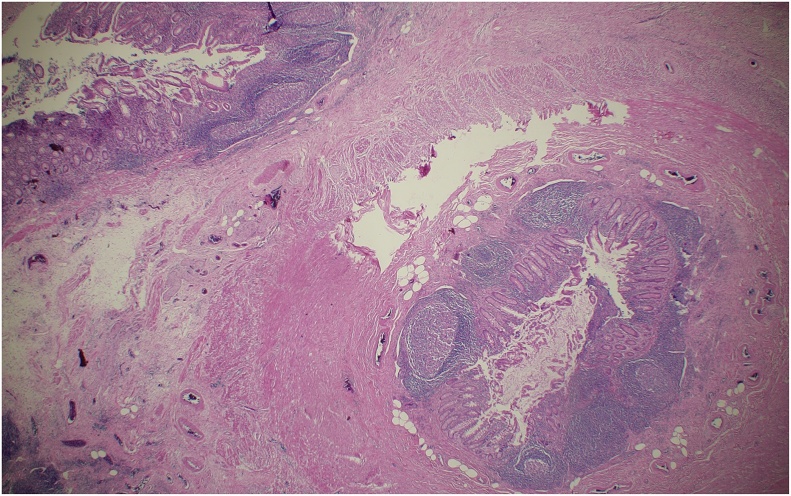
Appendicular stump layers: mucosa, lymphoid and muscularis.

The pathology reported an appendiceal stump which measured 2.5 × 1 cm, with local abscess and mucosal necrosis with transmural inflammatory infiltrate (Fig. 2). The patient developed an abdominal wall abscess with fascial necrosis and required three surgical interventions for drainage, debridement, and wall reconstruction. He was discharged home with a successful follow-up in ambulatory consult.

## 4 Literature review

### 4.1 General characteristics

We identified 97 articles which reported 131 cases of SA. We added our case to the database for a total of 132 cases (Table 1). In 126 genre was reported. Of them, 76 (60.3 %) were male. The age at SA was 33 years (IQR 24–53).

**Table 1:** Characteristics of patients with stump appendicitis.

Variable	No.	No. (%) or Median [IQR]
Genre	126	
Male No. (%)		76 (60.3 %)
Type of initial surgery	117	
Laparoscopic appendectomy No. (%)		55 (47 %)
Open appendectomy No. (%)		62 (53 %)
Complicated initial appendicitis No. (%)		16 (12.1 %)
Type of complications at initial appendicitis*	16	
Abscess No. (%)		5 (31 %)
Perforation No. (%)		10 (63 %)
Gangrene No. (%)		3 (18 %)
Age at initial appendicectomy Median [IQR]	116	25 [13–37]
Age at stump appendicitis Median [IQR]	126	33 [24–53]
Time from initial appendicectomy to stump appendicitis. Median [IQR]	122	20 [7–120]
Management of stump appendicitis	132	
Laparoscopic appendectomy No. (%)		41 (31.06 %)
Open appendectomy No. (%)		57 (43.18 %)
Initial laparoscopic with posterior conversion No. (%)		9 (6.82 %)
Appendectomy (no technique described) No. (%)		15 (11.36 %)
Antibiotics and non-operative management No. (%)		8 (6.06 %)
Management through colonoscopy No. (%)		1 (0.76 %)
Percutaneous drainage No. (%)		1 (0.76 %)
Complicated stump appendicitis No. (%)	132	67 (51 %)
Type of complications at stump appendicitis*	67	
Abscess No. (%)		38 (56.7 %)
Perforation No. (%)		36 (53.7 %)
Gangrene No. (%)		10 (14.9 %)
Chronic symptoms No. (%)	132	24 (18.2 %)
Length of the stump Median [IQR]	82	3 [2–4]

* Complications are non-exclusive.

### 4.2 Initial AA

The median age at initial appendectomy was 25 years (IQR 13–37). In 15 cases the information of the surgical technique used in the initial procedure was not addressed, but of the remaining 117 cases, 53 % underwent an open appendectomy and 47 % had a laparoscopic procedure. Only 16 procedures were reported as complicated appendicitis, being the most frequent perforation in 10 (63 %) of the cases.

Time interval between the initial episode of appendicitis and SA, ranged from 1 day to 60 years, with a median of 20 months (IQR 7–120 months). During this time, a total of 24 cases presented chronic symptoms, mostly complaining of RLQ abdominal pain.

### 4.3 Stump appendicitis presentation

The most common symptoms presented with the episode of SA are RLQ pain in 104 (78.8 %), leukocytosis in 73 (55.3 %), peritoneal signs in 69 (52.3 %), fever in 57 (43.2 %), nausea or vomiting in 42 (31.8 %) and migration of pain in 20 (15.2 %) (Table 2). Considering those six typical symptoms, in 41 cases (31.3 %) patients had four or more of them.

**Table 2:** Symptoms presented by patients with stump appendicitis.

Variable	No.	No. (%) or Median [IQR]
Symptoms at stump appendicitis presentation		
Epigastric pain No. (%)		9 (6.8 %)
Periumbilical pain No. (%)		11 (8.3 %)
Hypogastric pain No. (%)		3 (2.3 %)
RLQ pain No. (%)		104 (78.8 %)
RUQ pain No. (%)		3 (2.3 %)
Lower abdominal pain No. (%)		11 (8.3 %)
Diffuse pain No. (%)		13 (9.9 %)
Migration of pain No. (%)		20 (15.2 %)
Peritoneal signs No. (%)		69 (52.3 %)
Nauseas/Vomiting No. (%)		41 (31.1 %)
Diarrhea No. (%)		8 (6.1 %)
Fever No. (%)		57 (43.2 %)
Leukocytosis No. (%)		73 (55.3 %)
Hypotension No. (%)		1 (0.8 %)
Tachycardia No. (%)		20 (15.2 %)
Number of typical symptoms*	132	
0 No. (%)		9 (6.8 %)
1 No. (%)		21 (15.9 %)
2 No. (%)		23 (17.4 %)
3 No. (%)		37 (28.1 %)
4 No. (%)		26 (19.7 %)
5 No. (%)		14 (10.6 %)
6 No. (%)		2 (1.5 %)
Images	128	
No image No. (%)		9 (7%)
Ultrasound No. (%)		15 (11.7 %)
CT No. (%)		78 (61 %)
Ultrasound and CT No. (%)		26 (20.3 %)

* Typical symptoms are: RLQ pain, leukocytosis, peritoneal signs, fever, nauseas or vomiting and migration of pain.

Use of images was reported in 128 cases. Ultrasound (US) was used in 15 cases (11.7 %), CT in 78 (61 %) and both US and CT in 26 (20.3 %) for a total of 104 (81.3 %) CT and 41 (32 %) US.

Interestingly, of the 42 patients who presented with four or more typical symptoms, 2 (4.8 %) went to surgery without any image, 5 (11.9 %) had an abdominal US, 27 (64.3 %) CT and in 8 (19 %) cases US and CT before surgery.

Overall, appendectomy was performed in 122 (92.4 %) cases. Surgical technique was reported in 107 cases. Open appendectomy was performed in 66 (61.7 %). In 9 of them, the procedure was initially laparoscopic but had to be further converted to open. The remaining 41 (38.3 %) underwent a laparoscopic procedure. Surgical technique was not reported in 15 cases.

Of the remaining patients, eight were managed with antibiotics and non-operative management (NOM), one with percutaneous drainage and another-one through colonoscopic removal of the appendicolith.

A complicated SA was reported in 67 (51.0 %) cases, being abscess and perforation the most common ones with 38 (56.7 %) and 36 (53.7 %) of patients respectively.

Last but not least, from the 81 cases where the length of the stump is described, the median length was 3 cm with an IRQ between 2–4 cm.

### 4.4 Incidence

We found three articles that gave us information enough to calculate an incidence. The first, by Mangi A.A. and Berger D.L. in 2000 [10]. They reported 2185 appendectomies and 3 cases of SA, for an incidence of 1.37 cases in 1.000. The second by Buttrick S.S. and coworkers in 2012 [11], with a total of 3252 appendectomies and 2 cases, for an incidence of 0.62 cases in 1.000. The third one by Dikicier E. et al. in 2018 [12], with 3130 appendectomies and 4 cases, for an incidence of 1.27 cases in 1.000. In our institution, we have performed a total of 4522 appendectomies and 1 case of SA for an incidence of 0.22 cases in 1000 (Table 3).

**Table 3:** Incidence of stump appendicitis.

Name	Year	Number of appendectomies	Number of stump appendicitis	Relation between stump appendicitis and appendectomies
Abeel A. Mangi	2000	2185	3	1.37 in every 1000
Simon S. Buttrick	2012	3252	2	0.62 in every 1000
Enis Dikicier	2018	3130	4	1.27 in every 1000
Current research	2019	4522	1	0.22 in every 1000

## 5 Discussion

A known, but rare complication from appendectomy after an episode of AA is SA. It is usually underrated, and the risk factors in developing this condition are not clear. Some authors suggested that an initial laparoscopic appendicectomy could associate to an increased risk since the junction between the appendix and the cecum might not be well visualized due to its two-dimension format or the inability to feel the characteristics of the appendix and cecum, that help determine the placement of the junction [13,14].

Other authors suggested that an initially complicated appendectomy could be a contributing factor since it might difficult the dissection and the visualization of the base [15]. However, this statement has not been supported, perhaps because it is unusual at the time of the case report to have the information on the initial episode of AA, especially in those in which the interval between the episodes is wide. In the collected series, only 38 cases reported this information, from which 16 complicated initial appendicitis was informed. Unfortunately, not any conclusion can be driven by this data.

In essence, both of these items relate to the fact that they might end up with a long appendiceal stump, being this the real factor that could be associated with SA. It is intriguing the lengths of the appendiceal stump shown in this series, with stumps as longs as 7 cm and a median of 3 cm. We consider they have a significant length, suggesting that perhaps, the first appendicectomy was poorly performed, therefore leaving a long stump which is probably more likely to get re-inflamed. Nevertheless, this is theoretically, since we can support this is clear evidence.

In the collected series, it is noteworthy the median and IQR between the two episodes of appendicitis of 20 months (IQR 7–120). It is striking the fact that 92.9 % required either US, CT, or both to perform the diagnosis. Furthermore, about half of all the cases had complicated SA (51 %), being mostly abscess and perforation. Besides, the primary symptoms seem to be very similar to the ones as a first episode of AA, about one-third presented with 4 or more of the symptoms to the emergency room. Of course, even though they might be considered to have a classical presentation of appendicitis, the fact that they all reported the previous appendectomy, practically eliminated this differential diagnosis because the incidence reported in the literature makes it seem so rare that makes it hard even to consider its possibility. Regardless, we believe the incidence of SA is not as rare as the one reported, since we calculated the incidence with data found in 3 articles and our own ranging from 0.22 to 1.37 in 1000 cases. All of them, higher than the 1 in 50.000 cases reported in the literature.

The risk factors for SA are not clear yet, but the data available suggest that a primary laparoscopic appendectomy is not related, and the length of the stump left in the first surgery might be associated with SA. About one-third present with clinical symptoms suggesting an appendiceal pathology and about 50 % end up being complicated appendicitis. The incidence of SA is higher than previously reported.

The main limitation we encountered was the lack of data available regarding the initial episode of acute appendicitis and its management, especially regarding the surgical technique, which might be a factor for consideration in future studies when available.

## Sources of funding

We have nothing to declare, no funding was necessary.

## Ethical approval

We have approval from the ethics committee of Fundación Valle del Lili.

## Consent

Patient consent form was sign by the patient.

## Author contribution

Daniela Burbano: data collection, data analysis or interpretation, writing the paper.

Alberto García: data analysis or interpretation, writing the paper.

Julián Chica: data analysis or interpretation, writing the paper.

Camilo Salazar: data collection, writing the paper.

Juan C Bravo: interpretation of pathology.

Juan S Toro: interpretation of image studies.

## Registration of research studies

This study didn’t require registration.

## Guarantor

Daniela Burbano and Alberto García.

## Provenance and peer review

Not commissioned, externally peer-reviewed.

## Declaration of Competing Interest

We have nothing to declare.
